# Long-term Psychological and Occupational Effects of Providing Hospital Healthcare during SARS Outbreak

**DOI:** 10.3201/eid1212.060584

**Published:** 2006-12

**Authors:** Robert G. Maunder, William J. Lancee, Kenneth E. Balderson, Jocelyn P. Bennett, Bjug Borgundvaag, Susan Evans, Christopher M.B. Fernandes, David S. Goldbloom, Mona Gupta, Jonathan J. Hunter, Linda McGillis Hall, Lynn M. Nagle, Clare Pain, Sonia S. Peczeniuk, Glenna Raymond, Nancy Read, Sean B. Rourke, Rosalie J. Steinberg, Thomas E. Stewart, Susan VanDeVelde Coke, Georgina G. Veldhorst, Donald A. Wasylenki

**Affiliations:** * Mount Sinai Hospital, Toronto, Ontario, Canada;; † University of Toronto, Toronto, Ontario, Canada;; ‡ Saint Michael's Hospital, Toronto, Ontario, Canada;; § The Scarborough Hospital, Toronto, Ontario, Canada;; ¶ Hamilton Health Sciences Centre, Hamilton, Ontario, Canada;; # McMaster University, Hamilton, Ontario, Canada;; ** Centre for Addiction and Mental Health, Toronto, Ontario, Canada;; †† Sunnybrook and Women's Health Sciences Centre, Toronto, Ontario, Canada;; ‡‡ Rouge Valley Health System, Toronto, Ontario, Canada;; §§ Whitby Mental Health Centre, Whitby, Canada;; ¶¶ North York General Hospital, Toronto, Ontario, Canada

**Keywords:** Severe Acute Respiratory Syndrome, Stress, Psychological, Health Personnel, Stress, Traumatic, Burnout, Professional, research

## Abstract

Healthcare workers (HCWs) found the 2003 outbreak of severe acute respiratory syndrome (SARS) to be stressful, but the long-term impact is not known. From 13 to 26 months after the SARS outbreak, 769 HCWs at 9 Toronto hospitals that treated SARS patients and 4 Hamilton hospitals that did not treat SARS patients completed a survey of several adverse outcomes. Toronto HCWs reported significantly higher levels of burnout (p = 0.019), psychological distress (p<0.001), and posttraumatic stress (p<0.001). Toronto workers were more likely to have reduced patient contact and work hours and to report behavioral consequences of stress. Variance in adverse outcomes was explained by a protective effect of the perceived adequacy of training and support and by a provocative effect of maladaptive coping style and other individual factors. The results reinforce the value of effective staff support and training in preparation for future outbreaks.

## Abstract 2

TOC Summary Line: Healthcare workers in hospitals affected by SARS experience increased psychological stress 1–2 years after the outbreak.

Severe acute respiratory syndrome (SARS) emerged from Guangdong Province, People's Republic of China, in November 2002 and spread rapidly; transmission occurred primarily in hospitals, often to healthcare workers (HCWs). Although initially virtually no literature was available to guide expectations of how an emerging infection would affect the psychological well-being of hospital staff (*1*), by the summer of 2003 the acute psychological impact of SARS had been widely studied. Significant emotional distress was present in 18%–57% of HCWs (*2**–**6*) and was associated with quarantine (*7*), fear of contagion (*6**,**8**,**9*), concern for family (*5**,**9**,**10*), job stress (*6**,**9*), interpersonal isolation (*6**,**9*), perceived stigma (*6**,**7**,**11*), conscription of nonspecialists into infectious disease work (*12*), and attachment insecurity (*10*).

Working in SARS-affected hospitals could have been traumatic for some HCWs (i.e., an event that "threatens an individual's life or physical integrity and involves a subjective response of fear, helplessness, or horror" [13]). Before the SARS coronavirus was identified (*14**–**17*), SARS was an infection of unknown cause, unknown mode of transmission, global spread, and high mortality, c haracteristics that generally increase perceived risk (*18*). However, although the SARS outbreak was acutely stressful, the longer term impact of SARS on HCWs is unknown.

Understanding the enduring occupational and psychological effects of working during this SARS outbreak is important because it involves the well-being of large numbers of HCWs. Additionally, this information has wider relevance to health systems in planning for emerging infections, including pandemic influenza (http://www.who.int/csr/disease/influenza/inforesources/en) and the potential for bioterrorism (*19*). Although healthcare work during the SARS outbreak and during an influenza pandemic will differ in key respects, SARS experience provides the most extensive information available about the effects on HCWs of a large-scale infectious outbreak. The objective of the Impact of SARS Study was to assess the long-term psychological and occupational impact of SARS outbreak on HCWs and to identify personal and systemic factors that increase vulnerability.

## Methods

### Design, Setting, and Participants

The study took place in hospitals in Toronto and Hamilton in Ontario, Canada. Most of Canada's 438 suspected and probable SARS cases were identified in Toronto. Hamilton HCWs were selected as a comparison group because Hamilton is 57 km from Toronto and experienced all of the healthcare processes and precautions associated with Ontario's response to SARS (e.g., restrictions on access to care, protocols for staff screening, isolation procedures) but did not have SARS patients. Hamilton and Toronto hospitals are otherwise similar in terms of size, workload, and organizational characteristics. Thirteen participating sites (9 Toronto, 4 Hamilton) included academic and community hospitals. All Toronto sites treated SARS patients. Eligible HCWs included nurses in medical and surgical inpatient units and all staff of intensive care units, emergency departments, and SARS isolation units. Fifty-five clinical units participated (Toronto 40, Hamilton 15) from October 23, 2004, to September 30, 2005. This study was approved by the Research Ethics Board of each hospital.

Survey A measured adverse outcomes. All participants completed survey A anonymously and received Can $10. Those who were willing to provide more information participated in survey B, which measured potential mediators of adverse outcomes, and in 2 structured interviews (results to be reported elsewhere). Participants in survey B also received $50.

A separate "representativeness survey" was conducted from September through November 2005 to compare eligible Toronto HCWs who had participated in the Impact of SARS Study with those who had not. HCWs were approached at staff meetings in 14 participating clinical units and asked to complete an anonymous, 6-question questionnaire that surveyed whether or not they had participated in the Impact of SARS Study, exposure to SARS patients, age range, job type, years of healthcare experience, and overall subjective impact of SARS on their lives.

### Measures

In the study instruments, "during the SARS outbreak" was defined for Toronto HCWs as the period from February 2003 to the day the last SARS patient was discharged from a participant's hospital or died. For Hamilton HCWs, the comparable period was defined as February through September 2003. SARS patients included probable and suspected SARS patients and persons isolated while their cases were under investigation for SARS according to the participants' report, rather than by using case definitions (http://www.phac-aspc.gc.ca/sars-sras/sarscasedef_e.html).

### Survey A

This survey measured demographic and job data as well as traumatic stress response (15-item Impact of Events Scale [IES] [20,21]), nonspecific psychological distress (Kessler Psychological Distress Scale [K10] [22]), and professional burnout (emotional exhaustion scale of the Maslach Burnout Inventory [MBI-EE] [23–25]). To measure the practical and functional impact of SARS experience, participants were surveyed about changes since the SARS outbreak in healthcare work hours and the amount of face-to-face contact with patients in their work. Survey A also asked if survey participants had experienced an increase since the SARS outbreak in smoking, drinking alcohol, or "other activities that could interfere with your work or relationships" and how many work shifts had been missed in the 4 months preceding the survey because of stress, illness, or fatigue.

### Survey B

Survey B, by using a previously described instrument, measured SARS-related perception of stigma and interpersonal avoidance; adequacy of training, protection, and support; and job stress (*6**,**10**,**26*). Scales calculated as the mean of all items related to these constructs showed adequate internal reliability (Table 1). Adaptive coping (problem-solving, seeking support, positive reappraisal) and maladaptive coping (escape-avoidance, self-blame, confrontative coping) regarding SARS were measured with the relevant subscales of the Ways of Coping Questionnaire (*27*), for which the stressful event was defined as the SARS outbreak. Attachment insecurity was measured with the anxiety and attachment avoidance scales of the Experiences in Close Relationships-Revised questionnaire (*28*).

**Table 1:** Scales to measure perceptions about severe acute respiratory syndrome (SARS) experience

Scale	Perception
Training, protection and support* Cronbach α = 0.89	I had adequate training to deal confidently with the situations that I faced.
	Infection control procedures were adequately explained.
	I received adequate training in infection control procedures.
	I was provided with the protective equipment and procedures that I needed.
	I had someone to ask when I had problems using equipment.
	The hospital where I worked took my well-being into account when decisions were made that affected me.
	Emotional support (e.g., counseling) was available to those who needed help.
	I felt appreciated by the hospital/clinic/my employer.
	My hospital/workplace was supportive.
Job stress† Cronbach α = 0.76	There was more conflict among colleagues at work.
	I felt more stressed at work.
	I had to do work that normally I don't do.
	I had an increase workload.
	I had to work overtime.
Perceived stigma and interpersonal avoidance† Cronbach α = 0.77	I thought that people avoided me because of my profession.
	I thought that people avoided my family members because of my profession.
	I coped with the SARS situation by avoiding crowded places.
	I coped with the SARS situation by avoiding colleagues who might be exposed.

*Items scored on a 5-point scale from 1 (very confident that this is false) to 5 (very confident that this is true). †Items scored on a 6-point scale from 1 (strongly disagree) to 6 (strongly agree).

### Statistical Analysis

Central tendencies of parametric variables are described by mean and standard deviation; nonparametric variables are described by median and interquartile range. Between-group differences in parametric variables were determined by Student t test and in nonparametric variables by Mann-Whitney U test. To make the identified between-city differences more clinically meaningful, the prevalence of high scores was determined with standard cutoff values: IES >26 (http://www.mardihorowitz.com), MBI-EE >27 (*25*), K10 >16 (http://www.crufad.unsw.edu.au). Between-group differences in categorical variables were tested by χ^2^.

To identify factors that might explain variance in adverse outcome, between-group differences in traumatic stress symptoms, psychological distress, and burnout were tested for the following categories: gender; duration of healthcare experience; job type; regular work during the SARS outbreak in emergency department, intensive care unit, or SARS isolation unit; indicators of the frequency and intensity of contact with SARS patients; and exposure to quarantine. A 10-day cutoff for quarantine was used, which corresponds to the standard period of quarantine for SARS (i.e., quarantine >10 days indicates extended quarantine or >1 period of quarantine). This analysis was performed in the full sample.

The relationship between adverse outcomes and potential mediating factors was identified by using Spearman rank-order correlations between adverse outcomes and measures of perceived systemic characteristics (stigma and interpersonal avoidance, adequacy of training, protection and support, and job stress) and psychological variables (coping style and attachment insecurity). This analysis was performed for survey A and B participants.

A stepwise regression analysis was performed for each adverse outcome. All potential mediating factors (those identified in the preceding univariate analyses with a significance of p<0.05) were entered. This analysis was performed for survey A and B participants.

Finally, to determine if factors that increase personal perceptions of risk had a practical functional impact on HCWs in the full sample, we identified an item in survey A that could serve as a proxy for the survey B factors that mediate vulnerability. This item is the duration (in months) of continuing perceived increased risk after the last SARS patient was discharged from a study participant's hospital or died. Duration of perceived risk was significantly correlated with the 2 SARS-specific mediating factors identified in the regression analysis: 1) maladaptive coping and perceived adequacy of training and 2) protection and support. For this analysis, the functional impact of SARS experience was operationalized as the number of adverse outcomes experienced by a person (from 0 to 7) of the following 7 outcomes: posttraumatic stress (IES >26); psychological distress (K10 >16); burnout (MBI-EE >27); decrease in face-to-face patient contact since SARS; decrease in work hours since SARS; increase in smoking, alcohol, or other problematic behavior since SARS; and >4 shifts missed because of stress or illness in the 4 months before the survey.

## Results

In total, 1,984 HCWs received detailed information about the Impact of SARS Study and 769 (39%) completed survey A. The interval between the last SARS patient discharged or deceased and study participation was 13–25 (median 19) months.

To determine how representative participants were of all eligible hospital staff, after the Impact of SARS Study a representativeness study was presented to 258 Toronto HCWs who had been eligible; it was completed by 255 (99%) of these HCWs. Exposure to SARS patients was more common in HCWs who participated in the Impact of SARS Study than those who did not. However, study participants and nonparticipants did not differ in age range, job type, years of healthcare experience, or overall subjective impact of SARS on their lives (Table 2).

**Table 2:** Comparison of eligible Toronto healthcare workers who chose to participate or not to participate in the Impact of SARS Study*

Characteristics	Participation in Impact of SARS Study		p value
	Did not participate, % (n = 144)	Participated, % (n = 111)	
Age group, y			
<40	53	44	
>40	47	56	0.17
Job type			
Nurse	73	71	
Other	27	29	0.76
Experience, y			
<10	51	41	
>10	49	59	0.12
Treated SARS patient			
Yes	31	59	
No or don't know	69	41	<0.001
Overall impact			
Bad	40	50	
Neutral or good	60	50	0.11

*SARS, severe acute respiratory syndrome.

Of the 769 participants, 73.5% were nurses (69.4% staff nurse, 3.1% manager or educator, 1.0% infection control practitioner). The next most prevalent job types were clerical staff (8.3%), physicians (2.9%), and respiratory therapists (2.3%). The remaining 99 participants (12.9%) were distributed among 14 different job types. Other characteristics of study participants, by city of employment, are presented in Table 3. Most Toronto participants (71.6%) reported contact with SARS patients, and Toronto participants were much more likely than Hamilton participants to have experienced quarantine (47.9% vs. 1.6%, p<0.001), which confirms the anticipated difference in SARS-related experience between comparison groups. A higher proportion of Hamilton participants were nurses (Hamilton 84.1% nurses vs. Toronto 71.2%, p = 0.001).

**Table 3:** Demographic and job characteristics of participants, Impact of SARS Study*

Characteristics	Toronto % (n = 587)	Hamilton % (n = 182)	p value
Female	86.0	89.6	0.22
Single	23.7	20.3	
Married or common-law	65.2	68.1	
Separated or widowed	11.1	11.5	0.41
Living with child <16 y of age	36.3	36.8	0.90
Living with adult >65 y of age	9.2	5.5	0.11
Worked in healthcare >10 y	65.1	68.7	0.37
Worked any shifts during SARS in			
Surgical inpatient unit	13.8	18.7	0.11
Medical inpatient unit	26.4	21.4	0.18
Isolation unit with SARS patients	22.5	†	
Intensive care unit	32.9	34.1	0.66
Emergency department	32.2	24.7	0.06

*SARS, severe acute respiratory syndrome. †Hamilton had no patients with SARS.

Survey B was completed by 187 HCWs (survey A and B participants). Survey A and B participants did not differ significantly from participants who only completed survey A by sex, job type (nurse or other), or city of employment. Survey A and B participants were older (mean 45 ± standard deviation 9 years vs. 41 ± 10 years, p<0.001) and more experienced in healthcare work (21 ± 10 years versus 16 ± 10 years, p<0.001). Survey A-only participants and Survey A and B participants did not differ with respect to exposure to SARS patients, working >5 shifts in intensive care unit, emergency department or SARS isolation unit during the outbreak or with respect to traumatic stress symptoms, psychological distress, or burnout.

During the study period (13–25 months after the SARS outbreak), Toronto HCWs reported significantly higher levels of burnout (Toronto median score 19, interquartile range 10–29; Hamilton 16, 9– 23, p = 0.019), psychological distress (Toronto 15, 12–19; Hamilton 13, 11–17, p<0.001), and posttraumatic stress (Toronto 11, 4–21; Hamilton 7, 0–19, p<0.001). To make these differences more clinically meaningful, the prevalence of high scores was calculated (Table 4). The prevalence of the following functional indicators of distress since the SARS outbreak was higher in Toronto HCWs: decrease in patient contact and work hours, increase in substance use and other traits that interfere with function, and more days off work (Table 4). Of the 7 adverse outcomes reported in Table 4, Toronto HCWs were more likely to be experiencing >1 problem (Toronto 68.1% vs. Hamilton 50.1%, p<0.001)) and were almost twice as likely to be experiencing multiple (>2) problems (Toronto 44.0% vs. Hamilton 22.5%, p<0.001).

**Table 4:** Prevalence of adverse outcomes in Hamilton and Toronto healthcare workers*

Adverse outcomes	Toronto, n = 587, %	Hamilton, n = 182, %	p value
High burnout (MBI-EE score >27)	30.4	19.2	0.003
High psychological distress (K10 score >16)	44.9	30.2	<0.001
High posttraumatic stress (IES score >26)	13.8	8.4	0.06
Since SARS have			
Decreased face-to-face patient contact	16.5	8.3	0.007
Decreased work hours	8.6	2.2	0.003
Increased smoking, drinking alcohol, or other behavior that could interfere with work or relationships	21.0	8.1	0.001
Missed >4 work shifts because of stress or illness	21.6%	12.6%	0.007

*MBI-EE, Maslach Burnout Inventory; K10, Kessler Psychological Distress Scale; IES, Impact of Events Scale; SARS, severe acute respiratory syndrome.

Personal and occupational characteristics of participants and the relationship of these variables to adverse outcomes are shown in Table 5 and Table 6. Univariate relationships significant at the level of p<0.05 were retained for stepwise regression analysis to determine which of these variables accounted for significant variance in each adverse outcome (Table 7). Maladaptive coping and perceived adequacy of training together with protection and support explained 18% of the variance in burnout. The same 2 variables explained 21% of the variance in posttraumatic stress. Maladaptive coping and attachment anxiety, together with a protective effect of experience in healthcare, explained 31% of the variance in psychological distress.

**Table 5:** Relationship of healthcare worker, job, and SARS exposure characteristics to adverse outcomes in Toronto healthcare workers*

Characteristics		Burnout				Psychological distress			Posttraumatic stress		
		n	Median	Interquartile range	p value	Median	Interquartile range	p value	Median	Interquartile range	p value
Sex											
	Male	82	18	9–29		14	12–19		10	2–19	
	Female	505	19	10–29	0.30	15	12–19	0.91	12	4–21	0.02
Job type											
	Nurse	418	21	11–29		14	11–18		12	5–22	
	Other	169	14	8–27	0.002	15	12–20	0.16	10	2–19	0.1
Healthcare experience											
	<10 y	205	21	12–30		16	12–21		11	11–21	
	>10 y	382	18	10–28	0.82	14	11–18	0.03	11	5–22	0.06
Worked on SARS unit											
	<5 shifts	498	19	10–30		15	12–19		12	4–22	
	>5 shifts	89	17	11–26	0.75	15	11–20	0.54	10	3–17	0.63
Worked in ICU											
	<5 shifts	427	20	10–30		15	12–19		11	4–21	
	>5 shifts	160	17	9–17	0.02	14	11–20	0.29	11	3–22	0.46
Worked in Emergency											
	<5 shifts	434	18	10–28		15	12–20		12	5–21	
	>5 shifts	153	21	10–32	0.12	13	11–17	0.005	9	2–21	0.24
Ever in SARS patient room											
	No	167	19	9–30		15	12–19		11	4–22	
	Yes	420	19	10–28	0.33	15	11–19	0.09	12	4–21	0.16
Touched SARS patient											
	No	265	19	9–30		15	11–19		12	4–22	
	Yes	322	19	11–28	0.42	15	12–19	0.32	11	4–22	0.41
Protected contact with saliva or phlegm of SARS patient											
	No	438	19	9–29		15	12–19		11	4–21	
	Yes	149	19	11–29	0.43	15	12–18	0.78	10	4–22	0.44
Unprotected exposure to SARS patient											
	No	502	18	9–28		15	11–19		11	4–21	
	Yes	85	24	13–32	0.012	16	13–22	0.08	13	6–22	0.38
In SARS patients' rooms >5 min, >5 times											
	No	316	18	9–28		15	11–18		11	3–21	
	Yes	271	20	11–31	0.08	15	12–21	0.02	11	5–22	0.24
Quarantined											
	Never	252	19	9–28		15	11–19		11	4–22	
	<10 d	235	17	10–28		15	11–19		11	3–21	
	>10 d	100	21	11–34	0.36	16	12–22	0.09	13	5–22	0.42

*SARS, severe acute respiratory syndrome.

**Table 6:** Correlation between adverse outcomes after SARS and perceived characteristics of workplace and environment, coping style, and attachment insecurity in Toronto healthcare workers*

Characteristics of healthcare workers	Burnout		Psychological distress		Posttraumatic stress	
	Spearman ρ	p value	Spearman ρ	p value	Spearman ρ	p value
Training, protection and support	–0.297	<0.001	–0.162	0.06	–0.269	0.001
Stigma and avoidance	0.153	0.07	0.080	0.36	0.302	<0.001
Job stress	0.312	<0.001	0.224	0.008	0.164	0.052
Adaptive coping	0.066	0.44	0.147	0.08	0.182	0.03
Maladaptive coping	0.261	0.002	0.312	<0.001	0.364	<0.001
Attachment anxiety	0.179	0.049	0.355	<0.001	0.295	0.001
Attachment avoidance	0.078	0.40	0.204	0.03	0.139	0.13

*SARS, severe acute respiratory syndrome.

**Table 7:** Variables that explain variance in adverse outcomes to severe acute respiratory syndrome (SARS) in Toronto healthcare workers

Variables	β	*t*	p value
Dependent variable: burnout*			
Maladaptive coping	0.29	3.34	0.001
Perceived adequacy of training, protection and support	–0.27	–3.10	0.002
Model R^2^ = 0.18, p<0.001			
Dependent variable: psychological distress†			
Maladaptive coping	0.31	3.78	<0.001
Years of healthcare experience	–0.26	–3.28	0.001
Attachment anxiety	0.24	2.87	0.005
Model R^2^ = 0.31, p<0.001			
Dependent variable: posttraumatic stress‡			
Maladaptive coping	0.37	4.39	<0.001
Perceived adequacy of training, protection and support	–0.22	–2.63	0.01
Model R^2^ = 0.21, p<0.001			

*Excluded variables: job stress, attachment anxiety, job type, worked in intensive care unit, unprotected contact with SARS patient(s). †Excluded variables: job stress, attachment avoidance, worked in emergency department, in SARS patients room >5 min or >5 times. ‡Excluded variables: perceived stigma and avoidance, adaptive coping, attachment anxiety, job type, sex.

Finally, the functional impact of vulnerability factors on the full survey A sample was tested by using duration of perceived risk after SARS as a proxy for the SARS-related vulnerability factors identified in the regression analysis. Duration of post-SARS perceived risk was correlated with maladaptive coping (Spearman ρ = 0.28, p = 0.001) and perceived adequacy of training, protection, and support (Spearman ρ = -0.27, p = 0.001). The Figure shows a linear increase in the prevalence of multiple adverse outcomes in HCWs with longer duration of perceived risk. Duration of perceived risk and the overall number of adverse outcomes were significantly correlated. (Spearman ρ = 0.23, p = 0.005).

**Figure:**
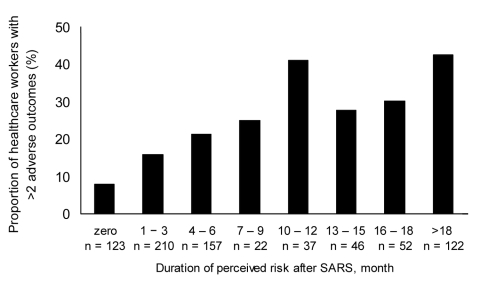
Relationship between prolonged perception of personal risk and reporting multiple adverse consequences of severe acute respiratory syndrome (SARS) in Toronto healthcare workers. Adverse outcomes are burnout; psychological distress; posttraumatic stress; decrease in face-to-face patient time since SARS; decrease in work hours since SARS; increase in smoking, drinking alcohol or other behavior that might interfere with work or relationships since SARS; and >4 work shifts missed because of stress or illness in the past 4 months.

## Discussion

This study highlights the resiliency of HCWs and, despite this trait, the potential that working during the SARS outbreak had a substantial negative impact on a statistically significant number of people. The evaluation of mediating factors suggests both systemic and individual targets for interventions to buffer the adverse effects of an extraordinary outbreak of infectious disease. Systemically, enhanced support and training may reduce burnout and posttraumatic stress. Individually, interventions that reduce maladaptive coping may decrease prolonged suffering.

The differences in adverse outcomes between Toronto and Hamilton HCWs were significant but small. However, further analysis suggests that the long-term impact of SARS has not been trivial. In particular, a categorical analysis (Table 4) shows that long-term adverse outcomes in Toronto HCWs occurred at a prevalence ≈50%–100% higher than in Hamilton HCWs. Furthermore, these outcomes may have a systemic impact, since SARS-affected HCWs reported reducing patient contact and hours of healthcare work as well as more frequent sick absences and an increase in behavior that could affect function.

These findings can be framed in terms of their potential value for the future. If the emergence of a new infectious disease is likely to increase the prevalence of significant distress in HCWs by 50%, to double the number of HCWs who are reducing their clinical practice or calling in sick, and if these difficulties will persist for >1–2 years after the outbreak's resolution, we want to learn from the SARS experience to try to buffer this negative impact. This discussion, therefore, addresses the identified mediators of SARS-related distress in HCWs and how these can guide preparation for pandemic influenza and other infectious disease outbreaks.

Exposure to high-intensity and high-risk work settings (such as intensive care units and emergency department work) and direct exposure to infected patients were not the primary determinants of adverse psychological outcomes. In fact, trends toward lower burnout in intensive care unit workers and less general psychological distress in emergency department workers were noted. These trends may be explained by the resilience of HCWs who choose this type of work and are consistent with the findings that longer healthcare experience was protective. We also found that the extent of various forms of distress was increased in Toronto HCWs, irrespective of their degree of contact with SARS patients, which implies that factors that are associated with the hospital environment as a whole and healthcare work in general during the outbreak were provocative.

Both systemic and personal variables were associated with persisting distress. In contrast to studies of distress during and shortly after the SARS outbreak (*6**,**9**,**12*), job stress related to conflict, workload, and conscription to new duties did not mediate long-term outcome. However, perceived adequacy of training, moral support, and protection were associated with better outcome. When the lessons of SARS are applied to pandemic planning, effective staff support may be a primary target to bolster the resilience of HCWs who will face future outbreaks. This observation is consistent with ones made during the SARS outbreak regarding the benefits of responsive communication (*29*), opportunities for facilitated reflection on normal emotional responses to extraordinary stress, and opportunities for HCWs to contribute to decision-making in the workplace (*10**,**30*).

Effective support benefits from careful planning and preparation before an outbreak, which the SARS situation did not allow. For example, effective moral or psychological support typically occurs in the context of trusted professional and institutional relationships, which should ideally be established before the outbreak situation. In particular, burnout has been identified as 1 of the most substantial health-related problems facing nurses (*31*). Because future outbreaks are likely to increase job strain and burnout, the prepandemic period is a critical time to attend to organizational characteristics that are known to buffer burnout, which include reducing patient-to-nurse ratios (*32*) and increasing organizational characteristics that increase nurses' autonomy, flexibility, control over practice (*33*), and perceived empowerment (*34*). The results of our study suggest that supportive interventions may be especially important for HCWs with fewer years of experience, who were more likely to experience prolonged psychological distress. Opportunities for mentorship or "buddying" with more experienced colleagues may be useful (*35*).

The personal variables that contributed to adverse outcomes were maladaptive coping through avoidance, hostile confrontation, and self-blame, and in the instance of general psychological distress, attachment anxiety. Although a review of interventions to modify coping style is beyond the scope of this paper, we note that organizational approaches to support staff and the individual experience of workers coping with extraordinary events are related. Hospital-based interventions to support staff may also promote adaptive coping. For example, engaging staff in collaborative planning for future outbreaks may reduce the tendency to cope by means of avoidant strategies and may enhance coping through problem-solving and peer-support. Anger and blame directed toward others (hostile confrontation) or oneself (self-blame) may be reduced in a working environment that fosters positive working relationships through effective leadership (*36*). Attachment anxiety is a common, relatively enduring, and stable interpersonal style within close relationships (*37*), which is known to be associated with sensitivity to stress under many conditions (*38**,**39*). Attachment anxiety is probably not a sensible target for hospital-based interventions to buffer the impact of systemic stresses, but it is a marker of those at greater risk for general psychological distress.

The results of this study also have implications for mitigating the effects of an infectious outbreak in the postoutbreak period. Because the duration of perceived risk in HCWs after the resolution of SARS is correlated with the severity of outcome, identifying and supporting HCWs who are at the highest risk for multiple and persistent psychological and occupational consequences of an outbreak may be possible by identifying HCWs whose perceived risk has not returned to normal within a few months after the event. Support programs, it would appear, need to be longer term to deal with ongoing residual effects after an outbreak. Programs directed toward healthy lifestyles, diet, exercise, and smoking cessation may also be important after the occurrence of an outbreak such as SARS to provide support to staff. Furthermore, for pandemic planning, the likelihood of prolonged subjective distress in a substantial percentage of HCWs should be factored into surge capacity modeling during and after the pandemic, particularly because distress is associated with reduced healthcare work.

Our conclusions are limited by the study method. With respect to generalizability, despite a response rate of 39%, the representativeness survey suggests that HCWs who participated were similar to nonparticipants. HCWs who had contact with SARS patients are overrepresented in the study sample, which may be because the study had greater salience for those persons, but study participants and nonparticipants did not differ in the subjective impact attributed to the SARS experience. A further limitation is that self-reports of SARS experiences do not provide an objective evaluation of actual differences in the training, protection, or support that HCWs received. Regardless of the limitations, the Impact of SARS Study provides a window on the long-term effects of working during times of extraordinary infectious risk.
